# Rapid High-Level Production of Functional HIV Broadly Neutralizing Monoclonal Antibodies in Transient Plant Expression Systems

**DOI:** 10.1371/journal.pone.0058724

**Published:** 2013-03-22

**Authors:** Yvonne Rosenberg, Markus Sack, David Montefiori, Donald Forthal, Lingjun Mao, Segundo Hernandez -Abanto, Lori Urban, Gary Landucci, Rainer Fischer, Xiaoming Jiang

**Affiliations:** 1 PlantVax Corporation, Rockville, Maryland, United States of America; 2 Institute of Molecular Biotechnology, RTWH Aachen University, Aachen, Germany; 3 Department of Surgery, Duke University Medical Center, Durham, North Carolina, United States of America; 4 Division of Infectious Diseases, Department of Medicine, University of California Irvine, Irvine, California, United States of America; Massachusetts General Hospital, United States of America

## Abstract

Passive immunotherapy using anti-HIV broadly neutralizing monoclonal antibodies (mAbs) has shown promise as an HIV treatment, reducing mother-to-child-transmission (MTCT) of simian/human immunodeficiency virus (SHIV) in non-human primates and decreasing viral rebound in patients who ceased receiving anti-viral drugs. In addition, a cocktail of potent mAbs may be useful as mucosal microbicides and provide an effective therapy for post-exposure prophylaxis. However, even highly neutralizing HIV mAbs used today may lose their effectiveness if resistance occurs, requiring the rapid production of new or engineered mAbs on an ongoing basis in order to counteract the viral resistance or the spread of a certain HIV-1 clade in a particular region or patient. Plant-based expression systems are fast, inexpensive and scalable and are becoming increasingly popular for the production of proteins and monoclonal antibodies. In the present study, *Agrobacterium*-mediated transient transfection of plants, utilizing two species of *Nicotiana,* have been tested to rapidly produce high levels of an HIV 89.6PΔ140env and several well-studied anti-HIV neutralizing monoclonal antibodies (b12, 2G12, 2F5, 4E10, m43, VRC01) or a single chain antibody construct (m9), for evaluation in cell-based viral inhibition assays. The protein-A purified plant-derived antibodies were intact, efficiently bound HIV envelope, and were equivalent to, or in one case better than, their counterparts produced in mammalian CHO or HEK-293 cells in both neutralization and antibody dependent viral inhibition assays. These data indicate that transient plant-based transient expression systems are very adaptable and could rapidly generate high levels of newly identified functional recombinant HIV neutralizing antibodies when required. In addition, they warrant detailed cost-benefit analysis of prolonged incubation in plants to further increase mAb production.

## Introduction

The prevention of mother-to-child-transmission (MTCT) of HIV during pregnancy, birth, and lactation is a pressing global health dilemma. Without specific intervention, MTCT of HIV can reach a rate of 40%, causing infection of >750,000 babies worldwide [1]. While single-dose nevirapine treatment can significantly reduce this transmission rate, such drug therapy selects for drug-resistant variants in the majority of recipient mothers [2]. In the absence of an efficacious vaccine, and as an alternative to anti-retroviral drug treatments, initial passive immunotherapy with a small number of broadly neutralizing monoclonal antibodies (mAbs) has shown promise in reducing MTCT in non-human primates [3]–[8] **.** These findings are consistent with the lower MTCT incidence in humans, particularly intrapartum transmission, observed when maternal neutralizing Abs are high [9], [10]. Specifically, anti-HIV mAb cocktails have been shown to protect neonatal and adult macaques from oral and vaginal challenge with chimeric simian/human immunodeficiency virus (SHIV) [6]–[8] reduce viral rebound after termination of antiretroviral drug therapy [11], are currently being formulated for use as vaginal microbicides [12] and could find application for post-exposure prophylaxis/combination therapy.

More recently, the identification of highly potent, broadly neutralizing mAbs such as VRC01, PG9 and PG16 [13], [14] and many mAbs of the PGT series [15] (mAbs against the CD4 binding site and epitopes in the V1/V2 and other regions of the HIV envelope) have greatly advanced the possibility that these mAbs will be used clinically as therapeutic agents. However, anti-HIV antibody cocktails for prophylaxis and therapy will require multiple doses and, despite their demonstrated ability to neutralize diverse viral strains, may potentially lose their effectiveness if viral resistance develops. To be an effective and available therapy, mAbs will 1) have to be produced on a very large scale and 2) may need to be generated quickly on an on-going basis in order to counteract resistance, to stop the spread of a certain HIV-1 clade in a particular region or to treat breast-fed babies and women who have previously received other mAbs during multiple pregnancies.

While historically, most recombinant therapeutic mAbs have been produced in mammalian cells, these expression systems lack the adaptability and the speed of more recent plant expression systems. These advantages, in addition to inexpensive scaled-up productions costs, have led to the increasing use of plants for product development/protein engineering [16], [17] perhaps becoming the system of choice for time critical applications, especially in emergency response situations. Recently, a transgenic maize-derived HIV mAb 2G12 [18], [19], has successfully completed a clinical phase I study for vaginal application and a plant cell-derived recombinant glucocerebrosidase enzyme, developed by Protalix Biotherapeutics in Isreal, has recently received regulatory approval as a human treatment of Gaucher disease (www.protalix.com).

For the most part, production has relied on the generation of transgenic plants, which, at least initially, is very time consuming and often suffers from insufficient yields. However, recent innovative Agrobacterium-mediated transient plant expression systems using plant viral-based vectors (Magnifection) [20] as well as non-replicative decon-structed or deleted viral-based vectors (CPMV-HT) [21] have been shown to be both rapid and highly productive; producing as much as 200–500 mg/kg in 6 days. Recombinant proteins produced in plants are essentially indistinguishable from those in animals with respect to protein synthesis, secretion, chaperone-assisted protein folding, and post-translational modification, including the early stages of N-linked glycosylation. In addition, plants provide excellent model systems for controlling subcellular trafficking patterns of proteins to achieve different glycosylation profiles [22]. A concern for plant-derived glycoproteins is the non-human complex glycans appended during the passage through the Golgi-system. Plant glycans are not sialylated, they can contain a core α(1,3)-fucose that differs from the core α(1,6)-fucose found in human and CHO-derived proteins, and a plant-specific β(1,2)-xylose [16], [22]. However several approaches, using knock-out or RNA silencing of glycosyltransferase genes to eliminate plant-specific glycans [23] as well as targeted protein retention in the ER by a carboxyterminal KDEL tag to produce only oligomannose (OGM)-type glycoforms [24], have successfully produced plants expressing human-like recombinant glycoproteins.

The ability to generate and evaluate alternative glycosylation patterns may be critical to the optimization of mAb efficacy and safety since Fc-mediated effector cell functions, ie. antibody-dependent cell cytotoxicity (ADCC) and antibody dependent cell viral inhibition (ADCVI), have been shown to impact viral inhibition [25]. Moreover, Fc glycosylation pattern can have a substantial effect on Fc-mediated antibody functions. For example, mAbs lacking the core α(1,6)-fucose exhibit significantly increased ADCC and ADCVI activity in vitro [26], [27] although no increased protection by a vaginally applied non-fucosylated variant of IgG b12 was observed, indicating a reduced role for FcγRIIIA mediated function in the case of vaginal infection [28].

The present studies describe the rapid production and functional characterization of six broadly neutralizing anti-HIV mAbs using two transient expression systems: (i) *Agrobacterium-*mediated infiltration of Ab genes alone into *Nicotiana tabacum cv Petit Havana SR-1* (*Nt*) or (ii) an enhanced production method involving co-infiltration of the p19 suppressor of gene silencing with the Ab genes into *Nicotiana benthamiana* (*Nb*) [29]. The mAbs initially chosen for evaluation were well-characterized, broadly neutralizing IgG_1_ mAbs specific for various regions of the HIV envelope with proven therapeutic potential individually or in antibody cocktails in non-human primate studies and/or human clinical trials; b12, (overlapping CD4 binding site (CD4bs); 2F5, 4E10, m43, (gp41); 2G12, (a high mannose cluster in the C3-V4 region)**;** a single chain m9, (CD4-inducible) and VRC01 (CD4 bs) [13], [30]–[35]. Each mAb was produced with and without a C-terminal KDEL tag to obtain preparations having different N-glycosylation profiles and harvested at different times to assess expression levels and quality. In addition to the mAbs, plant-derived glycoforms of the HIV 89.6.P gp140ΔCFI envelope were produced and used in antigen-binding assays.

While HIV- neutralizing and ADCVI activities of the plant-derived glycoforms were similar in each of the transient system and shown to be equal to the CHO or HEK-293 counterparts, plant-derived b12 preparations exhibited increased lower IC50s than the control and differed significantly in its binding profile to soluble monomeric **^BaL^**CHO gp120 envelope using SPR analysis.

## Materials and Methods

### Plant Expression Vectors

The codon usage of the human kappa (AAA58989.1) and human constant IgG1 (AAC82527.1) domains were adapted to tobacco by introducing codons preferentially used in highly expressed tobacco genes. The constant domains were flanked by type-IIs restriction enzymes to allow seamless joining of the variable domains. The codon usage of the variable antibody domains were also modified as described above and flanked by restriction sites generating non-palindromic compatible ends with the target vectors. The recombinant antibody genes were then generated by ligating the *Bfu*AI digested variable domains with the *Bfu*AI digested vectors containing the respective constant domains. Constructs with a C-terminal SEKDEL tag were generated by deleting a *Bbs*I fragment. The plant expression vectors were generated by cloning the *Eco*RI - *Xba*I fragments into the T-DNA vector pTRA-k [36] and verified by sequencing. A pBIN plasmid containing the tomato bushy stunt virus p19 inhibitor of silencing (**^TBSV^**p19) was kindly provided by Dr. Ulrich Commandeur (RWTH Aachen University).

### Agrobacterium and Plant Cultivation

Transformation, selection and cultivation of *Agrobacterium tumefaciens* strain GV3101 (pMK90RK) and cultivation of tobacco plants in the greenhouse or in growth chambers was essentially performed as described previously [24]. Briefly, recombinant *Agrobacteria* were cultivated in YEB medium with appropriate antibiotics, resuspended in MS medium to OD_600 nm_ = 0.5–1 and infiltrated either by injection or vacuum using 6–8 week old plants or leaves. For co-infiltration experiments, equal amounts of *Agrobacteria* carrying the light chain, heavy chain and p19 expression plasmids were mixed immediately before use. After infiltration plants were incubated at 20°C with a 16/8 h day-night cycle for 3 to 16 days and either directly used or harvested and stored at −20°C. For initial screening using ELISA and Western blotting, six leaf discs (∼11 mg) were collected from different positions in the transfected leaves, minced with 200 ul PBS and centrifuged.

### Scale-up and Purification

Large scale transfections were done to purify 60∼100 milligrams of HIV mAbs by protein-A affinity chromatography. Briefly, 150–300 g of the infiltrated tobacco leaves were blended with 2 volumes (v/w) of extraction buffer (0.05 M NaH_2_PO_4_, 0.25 M NaCl, 0.005 M Na_2_S_2_O_5,_ 0.005 EDTA, pH 5.5), centrifuged at 10,000 rpm for 20 min at 4°C, the supernatant filtered with a coffee paper filter, and the pH adjusted to 7.0 with NaOH. The supernatant was then kept at 4°C for about 2 hr and centrifuged again at 10,000 rpm for 20 min at 4°C, filtered again and applied directly onto an equilibrated protein-A column (20078, PALL life Sciences, NY). Bound antibodies were eluted using 100 mM glycine pH 3.5 and immediately buffered with 1/10 vol. of 1 M sodium acetate pH 4.75. Purified antibodies were washed with PBS and concentrated by ultrafiltration (MW cut-off, 30 kDa) (UFC903008, Millipore, Ireland).

### Plant Expression and Purification of HIV gp140 env

The 89.6P gp140gp140ΔCFI gene for the production of plant-derived recombinant HIV-1 gp140 env was kindly provided by Dr. Gary Nabel (Vaccine Research Centre, NIH) and subcloned into the pTRA-k vector. Both KDEL-tagged (designated as 188) and non-tagged (188AH) glycoforms were produced. Transgenic SR1 tobacco plants were generated using this clone as previously described [37].

For purification, transgenic tobacco leaves were weighed, blended with 2 volumes (v/w) of PBS and centrifuged at 10,000 rpm for 20 min at 4°C. The pH of the extract was adjusted to 7.25 with NaOH, kept for 2 hr at 4°C and centrifuged again. Extracts were applied directly onto a PBS equilibrated *Galanthus nivalis* agglutinin (GNA) lectin column (L8775,Sigma Aldrich, MO) which was washed with PBS containing 1 M NaCl and eluted with 1.2 M methyl-α-mannopyraoside in PBS (pH 7.25). Fractions were tested for the presence of gp140 by dot blot using 2G12 antibody and the positive fractions pooled and applied onto a DEAE column (DFF100, Sigma Aldrich, MO) equilibrated with 75 mM NaCl, 20 mM Tris, pH8. After washing with buffer, the column was eluted with 300 mM NaCl, 20 mM Tris, pH8. gp140 positive fractions were pooled, concentrated with Amicon Ultra-15 (100 K MWCO) and characterized using SDS-PAGE and western blotting.

### ELISA

For direct ELISA assays, 96-well Immuno Module plates (Nalge Nunc ) were coated with either anti-human kappa LC (50 µl of 1 ug/ml) (SIGMA K3502) or purified plant-derived gp140-KDEL (1 ug/ml) and incubated for 2 hr at RT with serial dilutions of leaf extracts or purified plant- or CHO-derived mAbs. The control HEK-293 VRC01 was kindly provided by the VRC, NIH and the CHO-derived 2G12 and 2F5 reagents by Polymun, Austria. Wells were blocked with 5% (w/v) milk in PBST, washed with PBST, incubated with a 1/10,000 dilution of peroxidase-labeled goat anti-human IgG (Fc) (A0170, Sigma), and developed with tetramethylbenzidine (TMB) liquid substrate system (T0440, Sigma Chemical Co, MO). Reactions were stopped with 0.5 N H_2_SO_4_, and endpoints were determined at 450 nm using the SPECTRA max PLUS plate reader (Molecular Devices). Purified plant-derived 2G12 was used as a standard.

### Anti-KDEL Western Analysis

Plant extracts containing the KDEL forms of either mAbs, 89.6Pgp140 or CHO BAL gp120 were loaded onto 4–12% SDS-PAGE gels (Lonza, MD) electro-transferred to nitrocellulose membranes (GE Healthcare Life Sciences, NJ), blocked with 5% milk in PBST while shaking at RT for 2 hr, washed 3X with PBST, incubated with mouse anti-KDEL mAb (10C3) (SPA-827, Assay Designs, MI) 1∶2000 dilution with PBST, RT for 2 hr, washed 3X with PBST, incubated with anti-mouse-IgG-HRP (NA931V, GE health care) 1∶5000 dilution with PBST, RT for 1.5 hr, washed 5X with PBST, developed with TMB membrane peroxidase substrate (50-77-18, KPL, MD). For estimation of molecular weights, ProSieve color protein molecular weight marker (Lonza, MD) was used.

### Surface Plasmon Resonance Analysis (Biacore)

Binding assays were performed on a Biacore T200 instrument at 25°C using a protein-A capture assay and HBS-EP as running buffer [38]. Antibodies were diluted into running buffer to yield the same capture level and soluble monomeric **^BaL^**gp120 (was injected for 180 s and dissociation was recorded for 450 s. The protein-A surface was regenerated by a 60 s pulse with 30 mM HCl. For each antibody a buffer injection was used for double referencing. To allow for direct comparison, the antigen binding curves were normalized to the antibody capture levels. Binding curves for 2G12 and VRC01 preparations were fitted by a simple monovalent interaction model. Relative antigen binding activities were derived by dividing the fitted R**_max_** of the sample by the fitted R_max_ for the CHO- or HEK-derived reference antibody. Binding curves for b12 were complex and therefore not fitted. Relative b12 activities were derived using the binding signals at t = 175 s.

### HIV Neutralization Assay

Neutralization of ER-retained and secreted mAb glycoforms of plant-derived mAb and their CHO- and HEK-derived counterparts was measured as a function of reductions in Renilla luciferase (LucR) reporter gene expression after a single round of infection in TZM-bl cells [39] and after infection with HIV-1 Env.IMC.LucR viruses in A3R5 cells. A3R5 cells [40] were obtained from Drs. Jerome Kim and Robert McLinden at the US Medical HIV Research Program (MHRP). This is a human CD4^+^ lymphoblastoid cell line (CEM/A3.01) [41] that was engineered at the US MHRP to express CCR5. Infectious molecular clones of HIV-1 carrying the entire ectodomain of the virus of choice and a Tat-regulated LucR reporter gene [42] were obtained from Drs. Christina Ochsenbauer and John Kappes at the University of Alabama, Birmingham. Briefly, a dose of virus that generates approximately 50,000 relative luminescence units (RLU) after 4 days of infection was incubated with serial 3-fold dilutions of test sample in duplicate in a total volume of 150 µl for 1 hr at 37°C in 96-well flat-bottom culture plates. Exponentially dividing A3R5 cells (90,000 cells in 100 µl of growth medium containing 25 µg/ml DEAE dextran) were added to each well. One set of control wells received cells+virus (virus control) and another set received cells only (background control). After 4 days of incubation, 90 µl of culture fluid was removed from each well, and 75 µl of cell suspension was transferred to 96-well white solid plates (Costar) for measurements of luminescence using the ViviRen Live Cell Substrate as described by the supplier (Promega, WI). Luminescense was measured using a Victor3 luminometer (PerkinElmer, MA). Neutralization titers are the dilution at which RLU were reduced by 50% compared to virus control wells after subtraction of background RLUs. Assay stocks of Env.IMC.LucR viruses were prepared by transfection in 293T cells [42] and were titrated in A3R5 cells.

### ADCVI Activity Assay

ADCVI was measured as described previously [27], [43]. Briefly, CEM.NKr-CCR5 target cells were infected with the clade B isolates SHIV162P3 or with HIV-1US657 for 48 hours, after which, the target cells were washed to remove cell-free virus. Serial dilutions of antibodies and of human PBMC effector cells (to achieve an effector:target cell ratio of 10∶1) were next added. Seven days later, p27 or p24 was measured in supernatant fluid by ELISA, and the percentage inhibition relative to a negative control antibody was determined.

## Results

### Transient Plant Expression of anti-HIV Neutralizing MAbs

Antibody genes were generated by joining the synthetic variable domains to the constant domains of the κ-light or γ1-heavy chain using appropriately designed *Bfu*AI overhangs. The C-terminal SEKDEL tag was generated by a subsequent deletion of a *Bbs*I fragment. All constructs were verified by sequencing and subsequently cloned as *Eco*RI-*Xba*I fragments into the T-DNA plant expression vector pTRA-k. The relative amounts of the Agrobacteria suspensions for the heavy and light chain were adjusted to 1∶1.2 to compensate for an imbalance in H and L chains after purification. It should be noted that co-infiltration/co-transformation of genes may result in imbalanced expression of heavy and light chain and impact reproducibility, but can be overcome by combining the expression cassettes onto the same T-DNA.

Two transient expression systems employing *Agrobacterium*-mediated transfection were used to produce HIV mAbs. Initially, *N. tabacum* cv. Petit Havana SR1 (*Nt*) leaves were infiltrated with mixtures of Agrobacterium containing expression constructs for the heavy (H) and light (L) chains of each of the early mAb (2G12, 4E10, 2F5, b12 and m43) and a single construct for the single-chain antibody m9. In order to increase expression levels, mAbs b12, 2G12 and VRC01 were then produced in a more recently utilized *N. benthamiana* (Nb) plants infiltrated with an Agrobacterium-suspension also containing the pTRA plasmid, which expresses the p19 suppressor of silencing from tomato bushy stunt virus (**^TBSV^**p19). The latter three mAbs were chosen for scaled-up production because of their demonstrated efficacy; plant-derived b12 having lower IC50s than CHO-b12, 2G12 because of its ability to protect in vivo [44] despite a relatively high IC50 and the more recently identified VRC01 because of its very broad and potent neutralizing properties. To produce high mannose or complex glycoforms, mAbs were produced with and without a C-terminal KDEL endoplasmic reticulum retrieval sequence.

**Figure 1:**
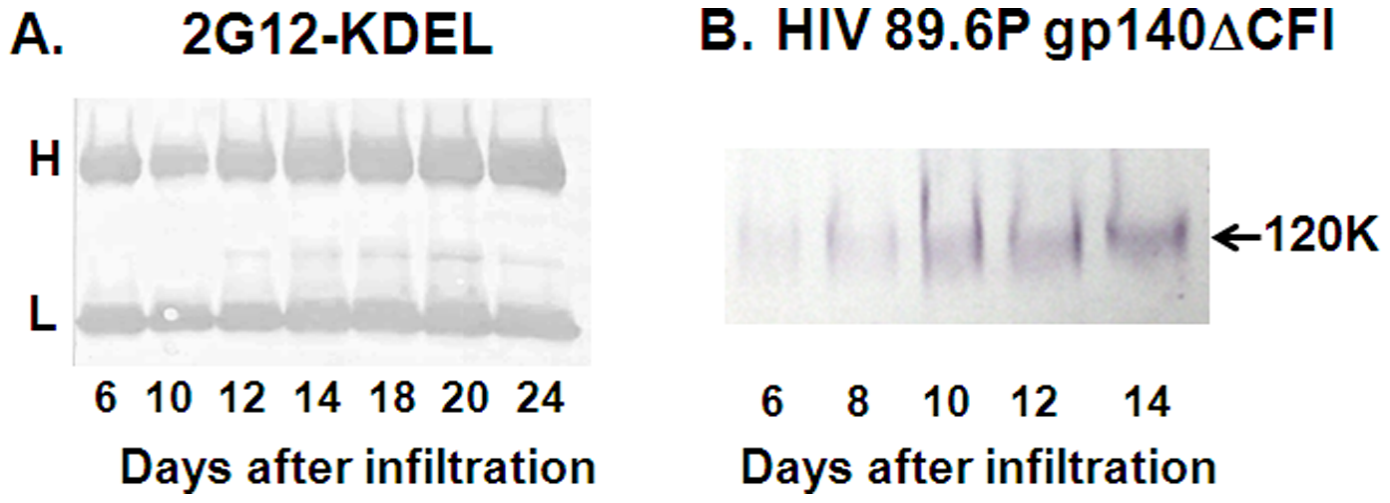
Accumulation of recombinant 2G12-KDEL and 89.6P gp140ΔCFI-KDEL env in leaves using the *Nb/*p19 transient plant expression system. Extracts produced from the equivalent of 3 mg (2G12-KDEL) (A) and 4.5 mg 89.6Pgp140ΔCFI env (B) of leaf biomass harvested from days 6–24 post-infiltration were loaded onto the gel and proteins detected by anti-KDEL Western blotting.

For initial screening, leaf disc extracts were tested by Western blotting using anti-KDEL antibody or by ELISA using anti-human kappa LC. Antibody accumulation in *N. tabacum* generally reached peak levels of 80–100 mg/kg at day 4 before declining (data not shown). By contrast, co-infiltration of *N. benthamiana* with the silencing inhibitor ^TBSV^p19 resulted in sustained and increased antibody synthesis with extended incubation times (Fig. 1), which is generally accepted to be due to a lack of post-transcriptional gene silencing (PTGS). In small scale experiments KDEL-tagged 2G12 accumulated to 100 mg/kg at day 6 post infiltration as measured by ELISA (data not shown) and further increased until day 18 after which the levels remained more or less constant (Fig. 1A). Similarly, VRC01 light chains showed sustained accumulation beyond day 6 post-infiltration; accumulating to ∼600 mg/kg determined semi-quantitatively by western blotting using purified VRC01-KDEL as standard (data not shown). A similar increase with time was also observed in the levels of 89.6P gp140 using the **^TBSV^**p19 system (Fig. 1B).

### N-glycosylation and Binding Properties of Plant-derived 89.6Pgp140ΔCFI

**Figure 2:**
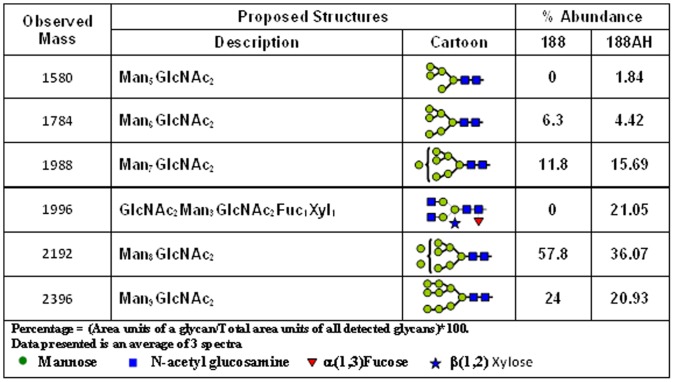
Comparison of glycan profiles of plant-derived 89.6Pgp140ΔCFI glyco-variants. MALDI TOF analysis was performed on the ER-retained 89.6P gp140-KDEL (#188) and the 89.6Pgp140 (#188AH) glycoproteins to determine the percentages of glycans present on each.

**Figure 3:**
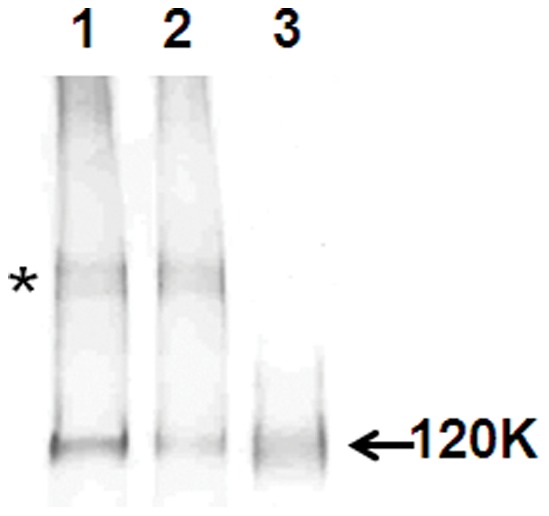
Western blotting analysis of purified plant-derived and CHO-derived HIV-1 env using 2G12. (lane 1∶89.6P gp140ΔCFI-KDEL (#188), lane 2∶89.6P gp140ΔCFI (#188AH) and lane 3: **^BAL^**CHO gp120. 200 ng of each sample was loaded.

KDEL-tagged (#188) and non-tagged variants (#188-AH) of HIV 89.6P gp140ΔCFI-env were stably produced in transgenic plants as well as in the transient Nb/p19 system (∼80 mg/kg). MALDI-TOF analysis confirmed that the ER-retained form contained only OMT glycans but also highlighted the low percentages of complex-type N-glycans (21%) on gp140 molecules not carrying a KDEL tag (Fig. 2). Western blotting analysis of the 188 and 188AH plant-derived glycoforms and the ^BAL^CHO gp120 env using 2G12 (Fig. 3) indicates that the plant derived forms have both monomeric and larger oligomeric forms (marked as asterisk Fig. 3), compared to the ^BAL^CHO gp120.

**Figure 4:**
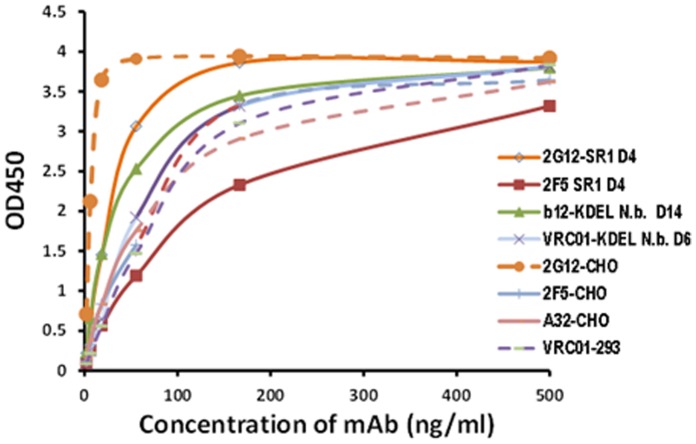
Comparison of binding of plant-derived HIV 2G12, b12, 2F5 and VRC01, CHO-derived 2G12, 2F5 and A32 and HEK-produced VRC01 to plant-derived 89.6P gp140ΔCFI-KDEL (#188) by direct ELISA. Purified plant-derived mAbs produced in SRI or *N.benthamiana* were harvested at the times indicated.

The purified 89.6Pgp140ΔCFI produced in plants was tested for binding to both plant-derived mAbs as well as CHO- or HEK-derived controls using a direct antigen ELISA (Fig. 4). All antibodies clearly recognized the recombinant plant-derived HIV envelope but differed in their reactivity. Plant- and CHO-derived 2G12, specific for high-mannose epitopes on the OGM 89.6Pgp140ΔCFI envelope, predictably exhibited the strongest binding.

### Antigen Binding Analysis of Plant-derived 2G12, VRC01 and b12 by Surface Plasmon Resonance (SPR)

**Figure 5:**
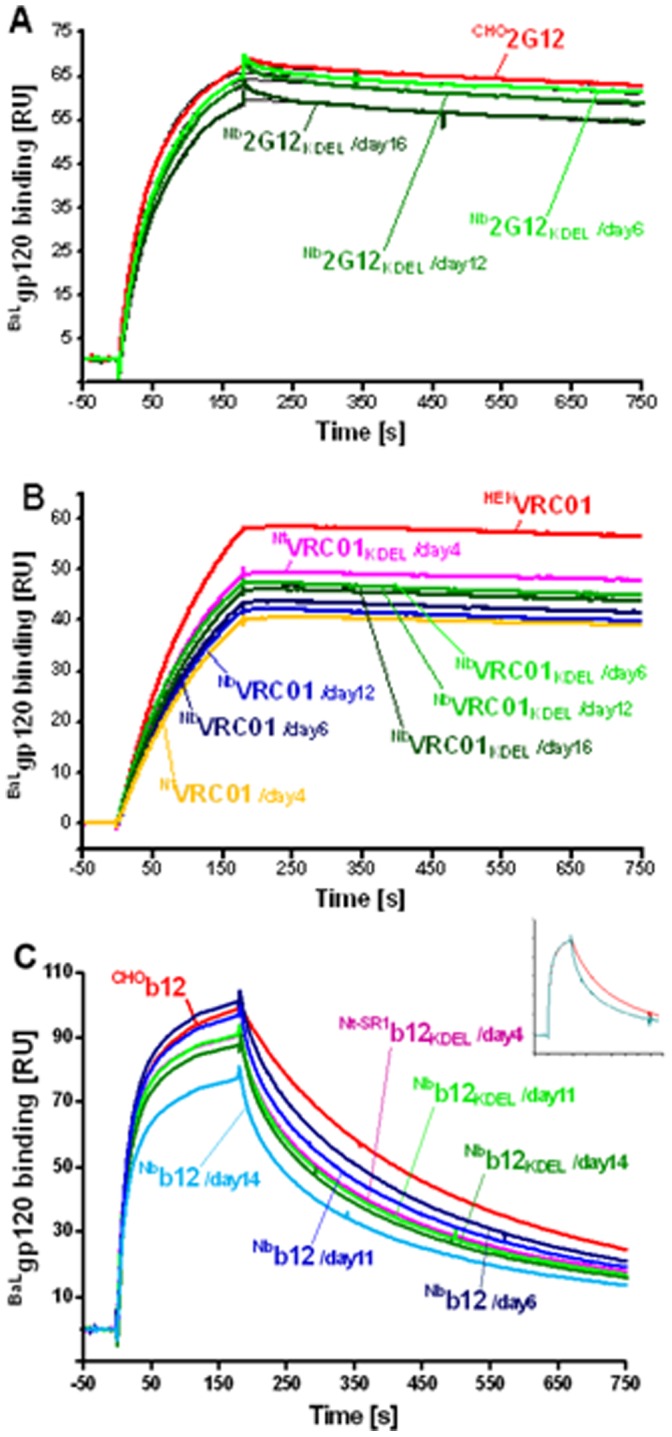
Binding of soluble ^BaL^CHO gp120 to protein-A captured 2G12(A), VRC01 (B) and b12 (C) mAb preparations by SPR analysis. ^BaL^CHO gp120 was injected at *t* = 0 for 180 s at a concentration of 100 nM, followed by a 450 second dissociation. Blank subtracted curves were normalized to the antibody capture levels for direct comparison. Insert: Normalized overlay plot for two CHO (red) selected b12 preparations to illustrate qualitative differences in the dissociation phase.

SPR was used to compare the binding of plant-derived 2G12, VRC01 and b12 preparations and their control CHO and HEK counterparts to soluble ^BAL^CHO gp120. Since the binding curves have been normalized to the antibody capture level, the curves (Fig. 5) and the fitted R**_max_** (Table 1) are directly comparable and reflect the antigen binding activity of the antibody preparations. There was an overall trend toward slightly lower activities following longer incubation times and slightly higher activity with KDEL tagged antibodies compared to non-KDEL tagged antibodies. As expected, all 2G12 and VRC01 preparations exhibited very similar binding kinetics and essentially differed only in their *R*_max_, i.e. antigen binding activity. In contrast, the binding curves of b12 antibodies differed considerably from 2G12 and VRC01 and could not be well described by a simple monovalent interaction, suggesting a more complex binding mechanism, probably involving an induced fit (Fig. 5). Thus, b12 preparations showed small but significant differences in their dissociation rate; exhibiting more pronounced biphasic dissociation with an increased fast component at later harvest times (D11 and D14> D6). This is easily visualized after scaling the two binding curves of **^CHO^**b12 and ^Nb^b12/day14) to the same binding level at the end of the injection period. The association phases entirely overlap whereas the dissociation phases are different (see insert, Fig. 5C). The reasons for this are not yet known, but are currently being investigated.

**Table 1:** Numerical results from the SPR analysis of HIV-BAL env binding.

Sample	Activity	*k*_ass_	*k*_diss_	*K*_D_	R_max_	Chi^2^
	%	[10^5^•M^−1^s^−1^]	[10^−4^. s^−1^]	[nM]	[RU]	
**^CHO^2G12** 1	108	1.2	1.03	0.86	74.7	1.42
**^CHO^2G12**	= 100	1.18	1.31	1.11	67.9	0.394
**^p19/Nb^2G12_KDEL_ day 6**	101	0.983	1.44	1.46	68.8	0.365
**^p19/Nb^2G12_KDEL_ day 12**	102	0.931	1.82	1.95	69.2	0.354
**^p19/Nb^2G12_KDEL_ day 16**	94	0.922	1.90	2.06	63.5	0.308
**^HEK^VRC01**	= 100	0.719	0.581	0.807	78.1	0.025
**^SR1−Nt^VRC01_KDEL_ day 4**	83	0.740	0.551	0.745	65.0	0.009
**^p19/Nb^VRC01_KDEL_ day 6**	83	0.699	0.884	1.27	65.2	0.008
**^p19/Nb^VRC01_KDEL_ day 12**	77	0.811	1.00	1.23	60.1	0.020
**^p19/Nb^VRC01_KDEL_ day 16**	80	0.698	0.959	1.37	62.70	0.012
**^SR1−Nt^VRC01 day 4**	75	0.618	0.715	1.16	58.60	0.022
**^p19/Nb^VRC01 day 6**	78	0.668	0.970	1.45	60.70	0.011
**^p19/Nb^VRC01 day12**	75	0.673	1.03	1.52	58.60	0.015
**^CHO^b12**	= 100	**N/A** 2				
**^SR1−Nt^b12_KDEL_ day 4**	92					
**^p19/Nb^b12_KDEL_ day 11**	92					
**^p19/Nb^b12_KDEL_ day 14**	89					
**^p19/Nb^b12 day 6**	103					
**^p19/Nb^b12 day 11**	98					
**^p19/Nb^b12 day 14**	78					

1 As reported [38], the comparison to these measurements shows an excellent reproducibility, with marginal differences being within experimental limits.

2 N/A. = not applicable.

### Neutralizing Activity of Plant-derived mAbs

**Figure 6:**
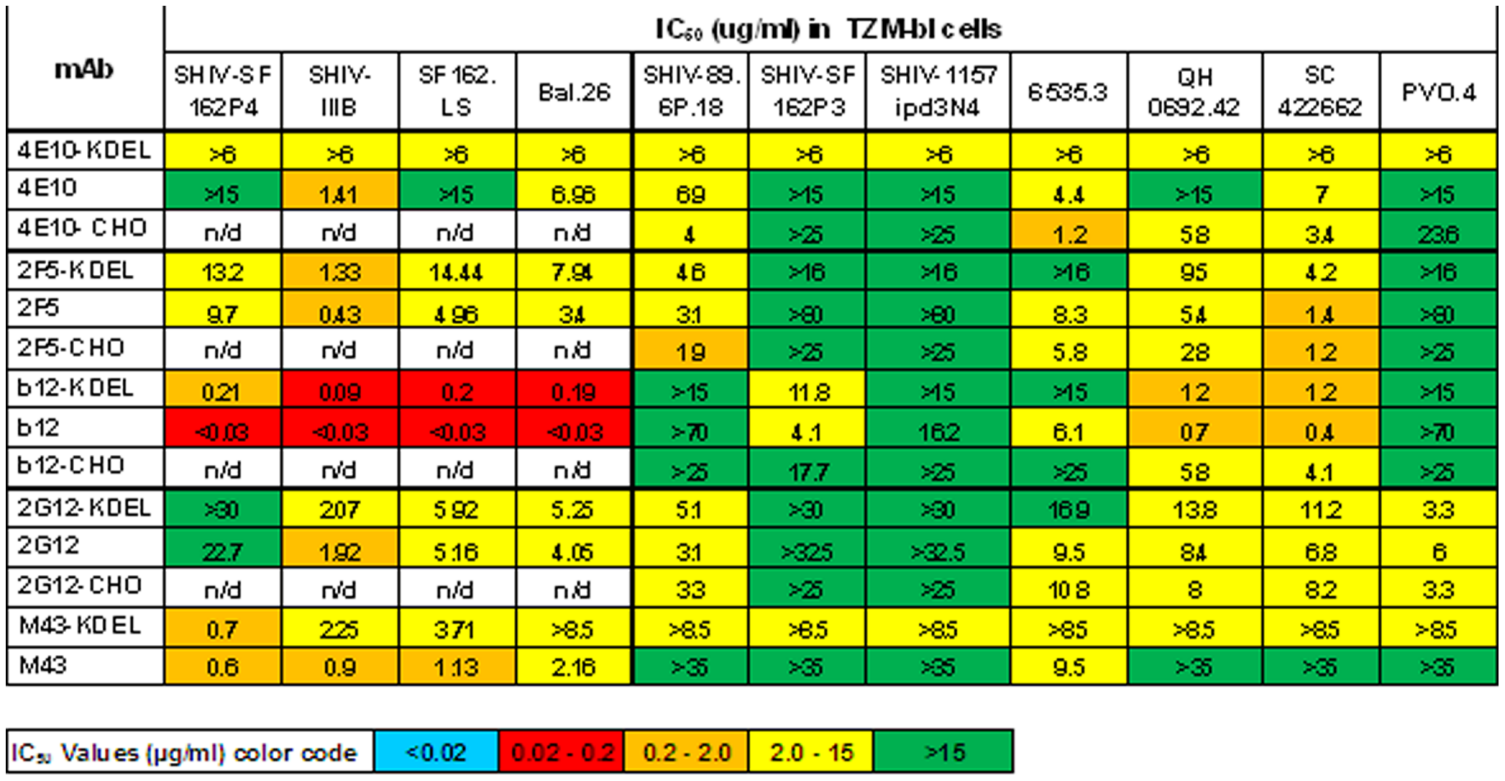
Comparison of the neutralization titers of *N.tabacum*-derived and CHO-derived mAbs using pseudovirus-based TZM-bl cells. All leaves were harvested at day 4. IC50s represent the concentration at which relative luminescence units (RLUs) are reduced by 50% versus virus control wells.

Neutralization activity of ER-retained OMT and secreted complex glycoforms of *N. tabacum* (SR1)-derived mAb harvested at day 4 was measured in a TMZ-bl assay as a reduction in Luc reporter gene expression after a single round of infection with a panel of Env-pseudotyped viruses. Initially neutralization activity of these mAbs was tested using a small panel of Tier 1 HIV-1 and simian/human HIV (SHIV) isolates (Fig. 6, left) and later compared with their CHO-derived mAb counterparts using a different panel of HIV isolates (Fig. 6, right). Comparison of the IC_50_s for plant-derived 2F5, 4E10 mAbs to those produced in the CHO and HEK cells demonstrate similar neutralization patterns. In general, except for 2G12, slightly higher IC50 values (i.e., less potent neutralizing activity) were observed with the secreted forms of the plant-derived antibodies, compared to those containing the KDEL ER retention signal. Importantly, the plant-derived b12 showed a 4–10-fold higher potency than the CHO-derived form against several HIV isolates. The IC**_50_** of the sc m9 antibody produced in plants was also similar to other CHO-derived mAbs in the HIV neutralization assay using TZM-bl cells and 2-fold better than a *E.coli*-derived form in a PBMC (Profectus Biosciences assay) (not shown).

**Figure 7:**
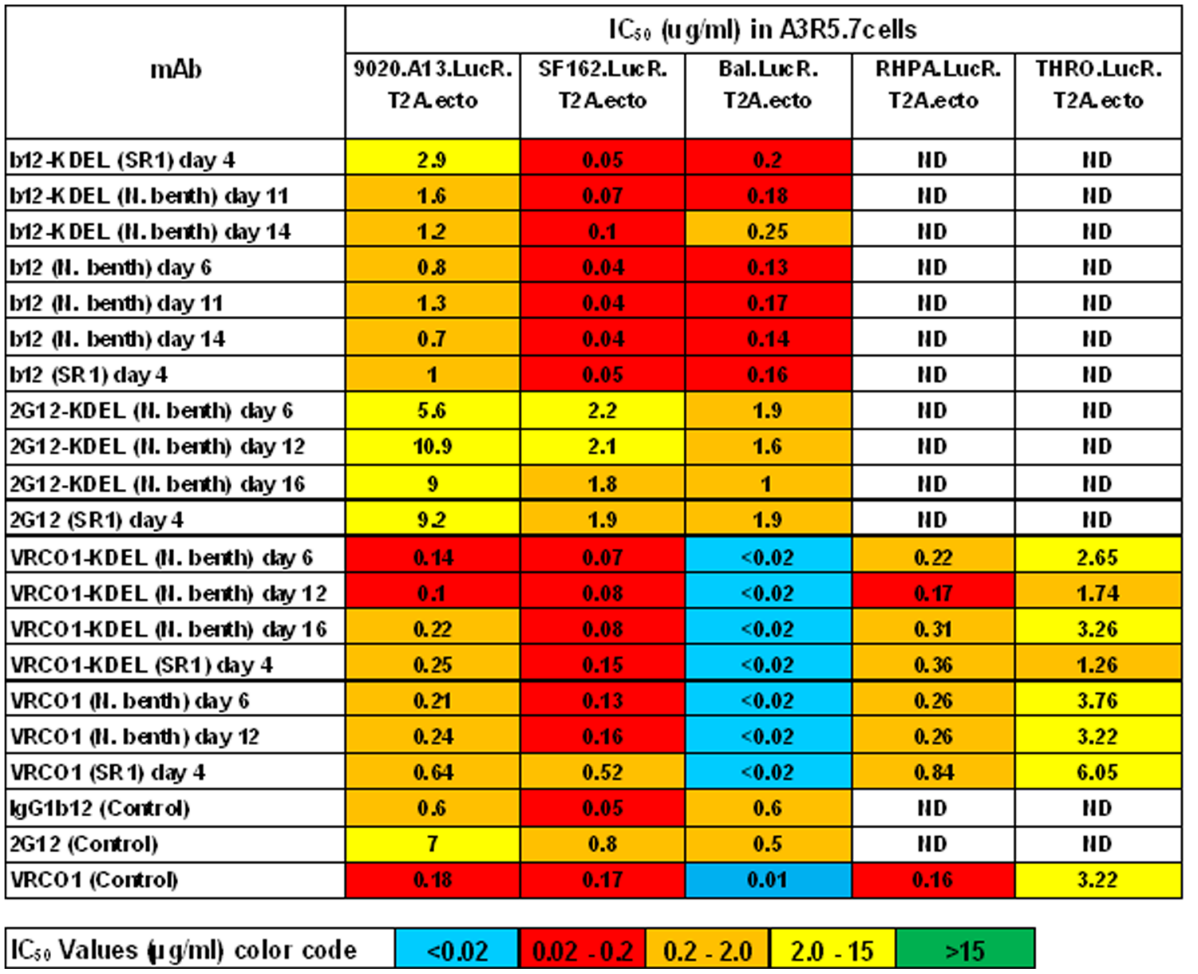
Comparison of the neutralization titers of *N.benthamiana*/p19-derived and control mAbs at different harvest times. IC50s represent the concentration at which relative luminescence units (RLUs) are reduced by 50% versus virus control wells. Times are indicated.

**Figure 8:**
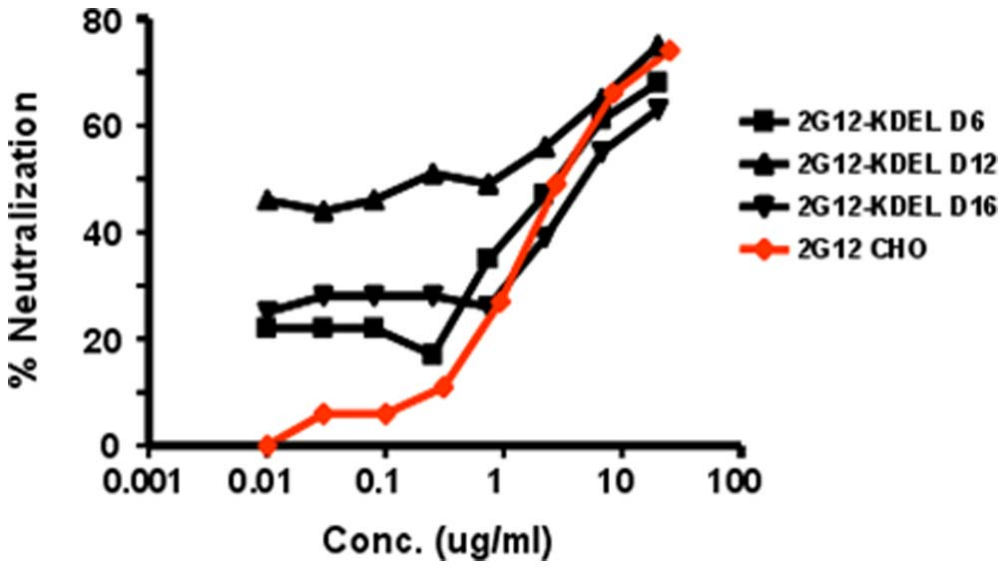
Titration of *N.benthamiana*/p19-derived 2G12 and CHO-derived 2G12 from different days of harvest in the TMZ-bl assay. Percent neutralization was assessed at various dilutions of CHO-derived 2G12 and purified plant-derived 2G12 extracted from leaves at days 6,12 and 16 post infiltration.

The IC_50_ values of the high mannose and secreted b12, VRC01 and 2G12 glycoforms harvested at different times in the *Nb/*p19 system were further assessed against a panel of Tier 1 HIV-1 isolates 9020.A13.LucR.T2A.ecto, SF162.LucR.T2A.ecto, and Bal.-LucR.T2A.ecto (Fig. 7). The IC**_50_** of these mAbs collected early (D6), mid (D12) or late (D14) post-infiltration are shown to be very similar, indicating that the longer incubation times and high level over expression in the leaves did not result in loss of HIV neutralizing activity. Interestingly, at low mAb concentrations of the D6, D12 and D16 2G12-KDEL samples, titration curves were quite different from each other and higher than the CHO-2G12 control (20–50% neutralization vs <10% respectively)(Fig. 8).

### ADCVI Activity of Plant-derived HIV mAbs

Historically, the ability of anti-HIV antibodies to bind envelope epitopes on cell-free virions and to inhibit their entry into CD4+ target cells has defined in vitro neutralization potency and thought to be predictive of in vivo protection. However more recently, evidence is accumulating that in addition to the requirement of high affinity antigen-Fab interactions, Fc-mediated activity may also play an important role.

**Figure 9:**
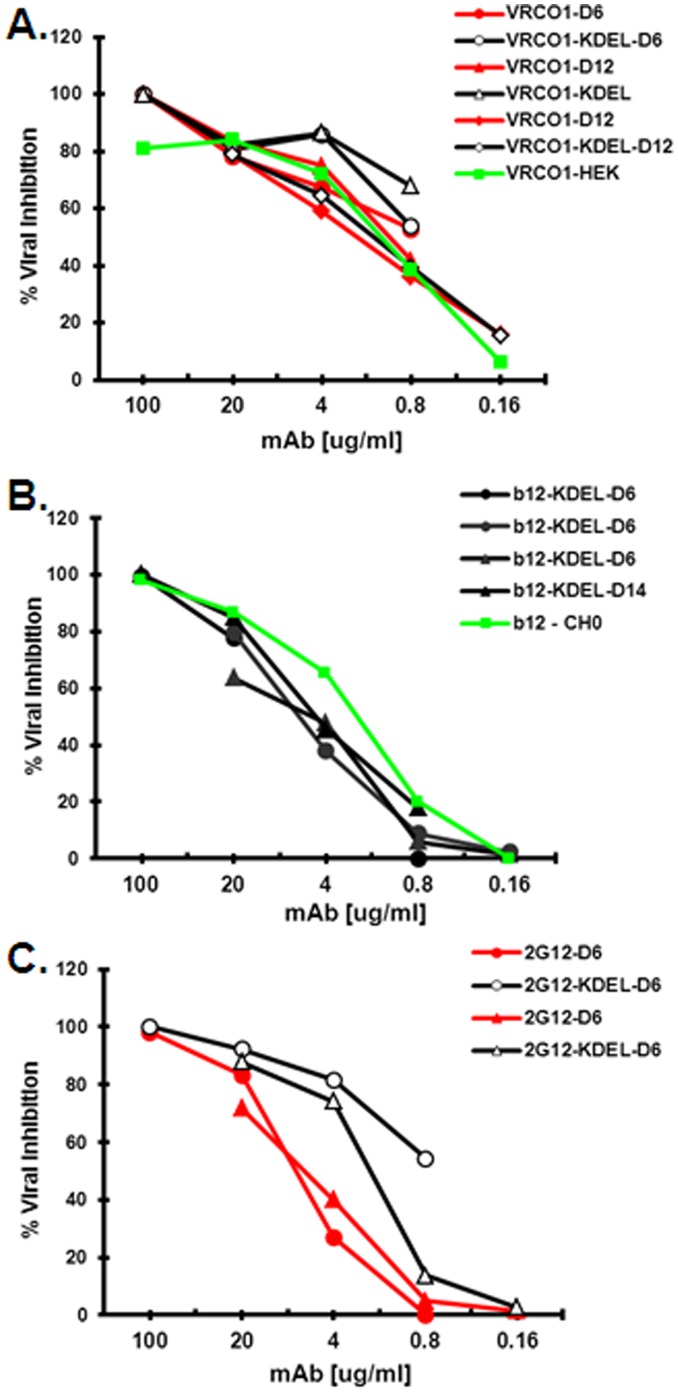
ADCVI activity of plant-derived glycovariants of VRC01 (A), b12 (B) and 2G12 (C) against SF162P3 and the US657 isolates. Black lines: OGM glycans (KDEL tag), red lines: complex glycoforms (non-KDEL), green lines: HEK-VRC01 and CHO-2G12.

To examine whether HIV mAbs produced in the transient plant expression system could exhibit Fc-mediated anti-viral activity, ADCVI assays were performed with the similar OGM and secreted glycol-variants of VRC01, 2G12 and b12 samples used in Table 1 and Fig. 8. The results indicate that, in the PBMC viral inhibition assays used, plant-derived glycoforms of VRC01, b12, and 2G12 exhibited similar activity as the VRC01-HEK and b12-CHO controls (Fig. 9A–C and ref. 27). In addition, while the levels of inhibition of the SF162P3 and US657 isolates by the KDEL-and non-KDEL forms of VRC01 overlapped, the OGM form of 2G12 appeared to have higher inhibitory activity against both the SF162P3 and the US657 isolates than the secreted complex glycoform consistent with the enhancement observed with α(1,3)-fucose-negative mAbs [27]. Overall, OGM and complex VRC01 glycoforms as well as the HEK control displayed 4–5-fold more activity than the b12 and 2G12.

## Discussion

Plant systems have many practical, economic, and safety advantages compared to more conventional production methods including mammalian, yeast, or bacterial cell cultures [16], [45]. Expression levels, binding properties and anti-HIV activity of a panel of well characterized full length IgG HIV neutralizing mAbs have been compared using two different rapid transient plant expression systems; short-term (day 4) production in *N. tabacum* generally resulting in mAb levels of 80–100 mg/kg of leaf biomass and long-term production in *Nb/*p19 (>6 days) utilizing the viral p19 suppressor of silencing protein resulting in enhanced expression levels from 200–500 mg/kg.

While plants are typically harvested at ∼day 6 using viral-based *N. benthamiana* systems such as Magnifection and CPMV-HT, the current studies have extended harvest times to increase yields. Thus, mAbs were extracted from leaves up to day 16 post agro-infiltration and examined for accumulation, quality and functional activity during p19-suppression of silencing; a concern being that prolonged incubation may lead to misfolded or poorly assembled mAbs and subsequent reduced activity.

In addition to HIV mAb production, different KDEL-tagged and non-tagged glycoforms of the HIV-1 89.6P gp140ΔCVI envelope (env) have been produced both transiently and in transgenic plants. A comparison of the N-glycosylation analysis by MALDI-TOF MS of each of the plant-derived gp140 glycoforms confirmed efficient KDEL-mediated ER retrieval of the 188 env (no complex glycans) and low (21%) complex type N-glycans on non-KDEL tagged gp140 (#188AH) molecules. This low percentage of complex glycans on the plant-derived env proteins, is consistent with the high mannose content of virion-associated HIV-1 env [46] and differs from the higher percentage of complex gylcans appended to recombinant env molecules produced in mammalian cell lines [47], [48].

The initial env binding analysis of the plant-derived mAbs by ELISA also used a KDEL-tagged plant derived gp140 (#188) harboring exclusively OMT N-glycans since these are known to be critical for the binding of N-glycan dependent antibodies such as 2G12 [33] and several new mAbs of the PGT series [15] and have been shown to be the env epitopes targeted by broad and potent neutralizing antibodies in humans [49]. While plant-derived 188 was recognized well by all mAbs tested, both CHO- and plant derived 2G12 predictably exhibited the highest reactivities to the OMT gp140 glycoform in direct ELISA assays. Similar results have been observed with glyco-engineered envelope containing high mannose glycans produced in yeast [50] and in 293T cells deficient in complex glycans due to an absence of glucosaminyltransferase I [46].

Binding properties of the plant-derived mAbs 2G12, b12 and VRC01 were further studied by SPR using monomeric CHO-produced ^BaL^gp120. Once again, the antigen binding kinetics of plant-derived 2G12 and VRC01 preparations were found indistinguishable from that of their mammalian counterparts; small differences in the specific antigen binding activity possibly being related to the presence of residual product-related impurities and easily removable by additional purification steps. In contrast, plant-derived b12 samples exhibited significant differences in their dissociation kinetics, with a more pronounced fast component compared to CHO-b12.

Consistent with their env binding properties, the IC_50_ neutralizing activity of the plant-derived VRC01 and 2G12 mAbs produced in both the *Nt* and *Nb*/p19 transient systems were similar to their CHO- and HEK-derived counterparts in TZM-bl- and A3R5- based assays. Importantly, b12 molecules exhibited the same pattern of neutralization as the CHO-derived forms usually with lower IC_50_s against the same Tier 1 and Tier 2 HIV isolates, even as late as D14 post-infiltration. Thus, whilst the reduced env binding of day 14–16 of 2G12, b12 and VRC01 samples by SPR analysis may reflect inefficient assembly, aggregates, inactivation, unfolding etc., their high potency (IC_50_∶0.04–0.7) indicates no reduction in neutralizing activity concomitant with the altered binding kinetics. The relative higher potency of 2G12-KDEL at low concentrations compared to CHO-2G12 was unexpected. However, higher potency of plant derived 2G12 has been observed previously [51], thought to be due to a higher proportion of 2G12 dimers and oligomers that have been shown to be more potent [52], [53] and possibly explaining the high in vivo activity of 2G12 [44]. It should be noted that while the panel of HIV isolates was not sufficiently extensive to assess the breadth of the neutralization response, several SHIV isolates were included to aid in selecting one appropriate for in vivo protection studies. Although CHO-derived b12 neutralizes only ∼50% of isolates compared to 90% with VRC01, plant-derived b12 is being more extensively studied because of unexpected glycan related activities unique to this mAb which have been observed in both the SPR and neutralization assays (in preparation).

No consistent difference in IC_50_ neutralizing activity was seen between the high mannose and complex glycoforms of the current plant-derived mAbs except for b12, where the non-KDEL form had reproducibly higher activity than the KDEL-form. In ADCVI assays, viral inhibition of the SF162P3 and US657 clade B isolates by plant- and CHO-derived VRC01 was 4–5 fold higher than the b12 or 2G12 and showed no clear distinction between the OGM and complex glycoforms. By contrast, the 2G12 results were consistent with previous findings showing that elimination of the α(1–3) fucose from the CH2 domain glycan of the KDEL-tagged molecules enhanced Fc-mediated anti-viral activity [26]. It should be noted that in addition to the unique properties of b12 glycoforms, the presence of a glycan in the VL chain of VRC01 has also been shown to alter certain properties of the plant-derived molecules (in preparation).

The 2G12, 2F5 and b12 neutralizing mAbs have previously been produced in transgenic maize [51] and tobacco plants [27], [54] as well as the BY2 tobacco suspension cultures [55] at lower expression levels than those observed in the current transient system utilizing the p19 suppressor of silencing protein but with similar IC50 [56]. In addition, preliminary glycan profiles of the mAbs produced in the current studies are shown to be similar to those described in a detailed glycan analysis of 2G12 produced using a non-replicative deconstructed or deleted viral-based CPMV-HT vectors [21].

In summary, the yields obtained using the *Nb/*p19 system are significantly higher than those previously reported in transgenic tobacco BY2 suspension cultures [36] or in time consuming and labor-intensive transgenic plants. This advantage, in the absence of any loss of function, clearly demonstrates the potential of plant-based transient expression systems for rapid production of large quantities of recombinant mAbs, especially in pandemic and emergency response situations where frequent and rapid production is required. In the case of HIV, the speed and efficiency of transient mAb expression systems are very well suited for developing potent antibody-based therapies that need to be generated quickly on an on-going basis to counteract viral resistance or the spread of a certain HIV-1 clade. The high functional activity in neutralization and ADCVI assays of the purified plant-mAbs harvested >7 days post-infiltration requires that a more detailed cost-benefit analysis of prolonged incubation for higher product production is warranted.
